# Goal conflict, goal facilitation, and health professionals' provision of physical activity advice in primary care: An exploratory prospective study

**DOI:** 10.1186/1748-5908-6-73

**Published:** 2011-07-15

**Authors:** Justin Presseau, Jill J Francis, Neil C Campbell, Falko F Sniehotta

**Affiliations:** 1 Institute of Health and Society, Baddiley-Clark Building, Richardson Road, Newcastle University, Newcastle Upon Tyne, NE2 4AX, UK; 2 Aberdeen Health Psychology Group and Health Services Research Unit, Health Sciences Building, University of Aberdeen, Foresterhill, Aberdeen, AB25 2ZD, UK; 3 Centre of Academic Primary Care, University of Aberdeen, Westburn Road, Foresterhill, Aberdeen, AB25 2AY, UK

## Abstract

**Background:**

The theory of planned behaviour has well-evidenced utility in predicting health professional behaviour, but focuses on a single behaviour isolated from the numerous potentially conflicting and facilitating goal-directed behaviours performed alongside. Goal conflict and goal facilitation may influence whether health professionals engage in guideline-recommended behaviours, and may supplement the predictive power of the theory of planned behaviour. We hypothesised that goal facilitation and goal conflict contribute to predicting primary care health professionals' provision of physical activity advice to patients with hypertension, over and above predictors of behaviour from the theory of planned behaviour.

**Methods:**

Using a prospective predictive design, at baseline we invited a random sample of 606 primary care health professionals from all primary care practices in NHS Grampian and NHS Tayside (Scotland) to complete postal questionnaires. Goal facilitation and goal conflict were measured alongside theory of planned behaviour constructs at baseline. At follow-up six months later, participants self-reported the number of patients, out of those seen in the preceding two weeks, to whom they provided physical activity advice.

**Results:**

Forty-four primary care physicians and nurses completed measures at both time points (7.3% response rate). Goal facilitation and goal conflict improved the prediction of behaviour, accounting for substantial additional variance (5.8% and 8.4%, respectively) in behaviour over and above intention and perceived behavioural control.

**Conclusions:**

Health professionals' provision of physical activity advice in primary care can be predicted by perceptions about how their conflicting and facilitating goal-directed behaviours help and hinder giving advice, over and above theory of planned behaviour constructs. Incorporating features of multiple goal pursuit into the theory of planned behaviour may help to better understand health professional behaviour.

## Background

Knowledge translation (KT), the application of research evidence into clinical practice, has been characterised as a haphazard process [1]. The KT process can be broken down into a series of behaviours performed by individuals to reach a goal (*i.e*., goal-directed behaviours, or GDBs). When viewed as such, theories of human behaviour can be employed to identify factors that predict the behaviours involved in translating research evidence into practice [2]. For example, clinical practice guidelines in the UK recommend that primary care health professionals provide all patients, and especially those at greater cardiovascular risk, with advice on engaging in regular physical activity (PA) for health promotion and disease prevention [3,4]. However, evidence suggests that provision of PA advice is less than optimal [5,6]. By acknowledging the provision of PA advice as a health professional behaviour, behavioural theory can be used to understand factors that account for variability in optimal PA advice provision.

Among theories of behaviour, the theory of planned behaviour (TPB) [7] has been tested across a variety of populations, behaviours, and contexts [8]. The TPB suggests that behaviour is a function of four constructs: intention, attitude (evaluation of the behaviour), subjective norm (perceived social pressures), and perceived behavioural control (PBC; ability). Intention, the key construct in the model, is a proximal predictor of behaviour as well as a mediator of the effect of attitude and subjective norm on behaviour and a partial mediator of the effect of PBC on behaviour. While the TPB is among the models with the best utility in predicting health professionals' GDBs [9,10], it is not without its limitations [11]. Among them is the issue of behavioural segregation: the TPB focuses on a single GDB, isolated from other GDBs engaged in by health professionals. In contexts of multiple goal pursuit, such as clinical consultations, these other GDBs may have a helpful or hindering influence on a focal GDB. Competition for limited resources (*e.g*., time, energy) may lead to goal conflict. However, engaging in some GDBs may be helpful and increase the likelihood that a particular GDB is performed, thereby representing goal facilitation. Goal conflict and goal facilitation may influence the extent to which a health professional engages in a given guideline-recommended behaviour. If so, the incorporation of these constructs into the behavioural pathway may supplement the explanatory power of the TPB and help to further understand KT processes. The current study aimed to explore whether goal conflict and goal facilitation are predictive of health professional behaviour beyond the proximal predictors of behaviour from the TPB.

The TPB has been frequently used to predict health professional behaviour. A systematic review of social cognition models applied to predict health professional behaviour identified 14 prospective studies testing the TPB with 1,882 health professionals [10]. The identified studies explained a frequency-weighted mean of 35% of the variance in health professional behaviour, and intention and PBC were each consistent predictors of behaviour [10]. Furthermore, when compared against other social cognition models within the same sample, the TPB is the most predictive model [9]. The TPB posits that while additional background constructs might be relevant to understanding behaviour, their effect should be mediated through the model [12]. Nevertheless, a number of other social cognitive constructs have been proposed to supplement the TPB. For example, Godin and colleagues [10] hypothesised an augmented TPB that includes additional predictors of intention (role and identity, moral norm, and health professional characteristics) and behaviour (habit and past behaviour). Although these constructs may increase the predictive utility of the model, they do not address the TPB's focus on a single GDB segregated from other concurrently pursued GDBs.

Clinical practice often involves health professionals performing numerous GDBs, each competing for limited resources in patient consultations, in particular time-related resources [13]. GDBs might conflict with (*i.e*., hinder) pursuing a particular GDB while other GDBs might create opportunities and be perceived to facilitate (*i.e*., help). Assessing perceptions about how conflicting and facilitating GDBs influence a focal GDB provides a way of accounting for the influence of the wider context of multiple goal pursuit which often characterises clinical practice. General medical practitioners perceive many of their GDBs as facilitating and conflicting with guideline-recommended GDBs such as prescribing to reduce blood pressure and providing PA-related advice. For example, GPs have reported that addressing the patient's agenda, treating acute illnesses, and prescribing to reduce cholesterol are among the GDBs perceived to conflict with giving PA advice [13]. Furthermore, taking a patient's history, addressing alcohol consumption and smoking, checking body mass index, and addressing well-being and stress are perceived by GPs to facilitate giving PA advice [13]. Thus, not only do health professionals engage in numerous behaviours, but many of these are also perceived as facilitating or conflicting.

It is not clear whether goal facilitation or goal conflict actually predict health professionals' behaviour beyond the predictive efficacy of leading social cognition models such as the TPB. However, evidence from other populations supports the potential of goal conflict and goal facilitation as predictors of health professional behaviour.

In other professional contexts, both goal facilitation and goal conflict have been shown to be associated with behaviour. In a management setting, goal conflict was negatively associated (medium effect size [14]) with attainment of a novel self-set goal four months later [15]. However, goal conflict was assessed on a bipolar scale ranging from instrumental (negative values) to conflicting (positive values) and the observed mean of 'goal conflict' was negative and within a range that would be considered as goal facilitation. The observed association may be more appropriately characterised as evidence of the relationship between goal facilitation and behaviour. In an academic context, university professors' conflict between teaching and research negatively predicted their research performance [16]. In a context of medical equipment sales, goal conflict was negatively associated with commitment and self-efficacy (conceptually similar constructs to intention and PBC in the TPB), and performance [17].

The relationship between goal facilitation and conflict and behaviour has also been investigated to further understand preventive health behaviour, such as participation in PA. Prospective studies predicting engagement in PA have demonstrated that goal facilitation, but not goal conflict, predicts PA beyond TPB constructs [18-20].

Goal conflict may be more readily perceived and predictive of behaviour when the conflicting GDBs under consideration are pursued within the same context as a focal GDB. Focusing on goal conflict perceived within a resource-constrained clinical setting may be a more appropriate test of the predictive utility of this goal construct. As such, the present study was interested in conflict and facilitation between a health professional's GDBs. We aimed to explore the predictive utility of goal facilitation and goal conflict in a health professional context. We hypothesised that goal facilitation and goal conflict would predict health professional behaviour over and above intention and PBC.

## Methods

### Participants

To our knowledge, the present study was the first to test goal conflict and goal facilitation as predictors of health professional behaviour in primary care. There was little existing evidence upon which to estimate the effect sizes for a formal power calculation, and thus this study was considered to be exploratory. We sent questionnaires to a random sample of health professionals from all 84 GP practices in NHS Grampian and all 69 practices in NHS Tayside, Scotland at baseline, targeting a final sample size of at least 157 health professionals. We estimated a 40% response at baseline and a 65% response at follow-up. Baseline questionnaires were sent to 606 health professionals (453 general practitioners, or GPs, and 153 nurses).

### Measures and data collection procedures

The focal goal-directed behaviour of interest in the current study concerned providing PA advice, a guideline-recommended behaviour [3]. Patients with hypertension have an elevated risk of cardiovascular disease, and increased PA is associated with a reduction in blood pressure [21]. The focal behaviour was specified as giving patients with an existing diagnosis of uncomplicated hypertension lifestyle advice for increasing their PA.

At baseline in March 2009, participants were sent a four-page postal questionnaire along with an invitation letter, an information sheet, an informed consent sheet, and a freepost return envelope. An identical follow-up questionnaire was sent to baseline respondents six months later, in October 2009 along with an invitation letter and follow-up reminders to non-respondents. This length of follow-up is consistent with previous research testing goal conflict and goal facilitation in other settings [18,19] and tests of the TPB in this population [22,23].

### Theory of planned behaviour

TPB constructs were measured at baseline using single items (to maximise response rates) in a single block prefaced with 'Please rate the following statements based on the following action: In the next two weeks, personally giving lifestyle advice for increasing physical activity to your patients with an existing diagnosis of uncomplicated hypertension.' Intention was measured with one item: 'I intend to do this' (1-strongly disagree to 7-strongly agree). PBC was measured with one item using a semantic differential scale: 'For me, doing this is...' (1-very difficult to 7- very easy). Attitude was also measured on a single semantic differential scale: 'For me to do this is...' (1-very bad practice to 7- very good practice). Subjective norm was assessed using one item: 'People whose opinion I value expect me to do this' (1-strongly disagree to 7-strongly agree).

### Goal facilitation and goal conflict

Measures for goal facilitation and goal conflict were adapted from existing scales [18,19] into two single items (to maximise response rates) and assessed at baseline. For goal facilitation, participants were asked to rate their agreement with the statement 'During these consultations, other things I do helpfully lead me to give lifestyle advice for increasing physical activity' on a scale ranging from 1-strongly disagree to 7-strongly agree. To measure goal conflict, participants were asked to indicate their agreement with the statement 'During these consultations, other things I do lead me to spend less time giving lifestyle advice for increasing physical activity' on a scale ranging from 1-strongly disagree to 7-strongly agree. Factor analytic and predictive evidence has shown that goal conflict and goal facilitation are best considered as independent constructs, and were therefore measured separately [18,19].

### Demographics

Participants were asked a series of demographic questions to assess their age, sex, graduation year, employment status (full-time or part-time) and role (GP or practice nurse).

### Behaviour

The behavioural outcome measure was administered at follow-up and consisted of two items. The first item asked participants 'How many patients with an existing diagnosis of uncomplicated hypertension have you personally seen in the past two weeks?' The second item asked 'and of those, for how many did you give lifestyle advice for increasing physical activity?' The outcome measure was computed as the proportion of patients to whom advice was provided, out of the patients with existing uncomplicated hypertension seen in the past two weeks.

### Ethics approval

Ethical approval for the current study was obtained from the North of Scotland Research Ethics Committee (REC No. 09/S0801/4).

## Results

### Participants

Sixty-nine of the 606 questionnaires sent at baseline were returned (11.4%), 53 of which were completed at follow-up (76.8%). At least one health professional from 57 of the 153 practices that were sent questionnaires at baseline responded (37.3%). Eight respondents were deleted list-wise for not having seen any patients with an existing diagnosis of hypertension in the past two weeks at baseline or follow-up. One participant was deleted list-wise due to missing data on predictor variables. The final sample comprised 44 primary care health professionals (37 GPs, 7 nurses) from 40 general practices. The cumulative response rate for the study was 7.3%. All nurses in the final sample were women, while 43% (16) of responding GPs were women. The population of GPs in NHS Grampian and Tayside from which participants were sampled was composed of a higher percentage of women (48%), indicating that the sample was slightly overrepresented by male GPs. Demographics are presented in Table 1.

**Table 1:** Correlations and descriptives of behaviour, social cognitions, goal facilitation and goal conflict (n = 44)

Variables	1	2	3	4	5	6	7	8	9	10	11	Mean	SD
1. Behaviour^1^	--											0.44	0.37
2. Intention^2^	0.50**	--										5.00	1.54
3. PBC^2^	0.29	0.48**	--									5.02	1.32
4. Attitude^2^	0.55**	0.62**	0.37*	--								5.64	1.20
5. Subjective norm^2^	0.27	0.42**	0.31*	0.52**	--							4.86	1.25
6. Goal facilitation^2^	0.50**	0.52**	0.49**	0.60**	0.44**	--						5.39	1.10
7. Goal conflict^2^	-0.43**	-0.15	-0.34**	-0.23	0.01	-0.20	--					4.02	1.61
8. Age (years)	0.16	0.01	-0.05	0.15	0.12	-0.10	-0.07	--				45.95	8.53
9. Graduation year	-0.16	-0.09	0.06	-0.09	-0.22	0.06	0.01	-0.94**	--			1986	9.35
												n (%)	
												
10. Sex	-0.16	-0.21	-0.02	-0.21	-0.15	-0.01	-0.10	0.26	-0.23	--		n = 23 (52%) women	
11. Occupation	0.58**	0.25	0.14	0.34*	0.15	0.19	-0.16	0.08	-0.06	-0.42**	--	37 GPs, 7 nurses	
12. Full- or part-time^3^	0.13	0.24	0.08	0.23	0.02	0.10	0.05	-0.18	0.16	-0.65**	0.26	n = 21 (48%) full-time	

**p<0.01; *p<0.05

^1 ^Proportion of patients provided with advice out of the number of patients seen in past two weeks

^2 ^7-point Likert scales, with higher scores representing agreement

^3 ^Five participants did not respond. Correlations with full- versus- part-time are based on n = 39

*Note*. PBC = Perceived behavioural control

### Drop-out analysis

Participants included in the final analysis were compared to those who did not respond to follow-up or were excluded. Participants did not differ significantly on any demographic variables or baseline predictor variables, except on attitude scores. Included participants (*M *= 5.64, *SD *= 1.20) had significantly (*p *= 0.007) lower attitude scores than those who were excluded (*M *= 6.33, *SD *= 0.80).

### Descriptive statistics and bivariate relations

Mean scores on intention, PBC, attitude, subjective norm, and goal facilitation were moderately positive. For goal conflict, some participants agreed that other GDBs they performed conflict with giving PA advice while others disagreed. Descriptive statistics and bivariate correlations between key study variables are presented in Table 1. Supporting TPB hypotheses, attitude, subjective norm, and PBC had medium-to-large correlations with intention [14]. Intention, attitude, goal facilitation, and goal conflict most strongly correlated with behaviour. Goal facilitation, but not goal conflict, was significantly correlated with intention. Goal facilitation and conflict were not significantly correlated with each other. Occupation was also strongly correlated with behaviour (with nurses more likely to give advice than GPs), supporting the idea of including occupation as a covariate in subsequent analyses.

### Goal conflict and goal facilitation as predictors of clinical behaviour

A hierarchical linear regression was conducted to test the hypothesis that goal facilitation and goal conflict predict health professional behaviour above and beyond intention and PBC. Intention, PBC, and occupation were entered at step one, and accounted for 47.7% of the variance in behaviour. As shown in Table 2, goal facilitation was entered at step two, and accounted for an additional 5.8% of the variance in behaviour (*p *= 0.034). Goal conflict was then entered in step three of the model, and accounted for an additional 8.4% of the variance in behaviour (*p *= 0.006). The final model showed that intention, occupation, goal conflict, and goal facilitation were each significant predictors of behaviour.

**Table 2:** Goal conflict, goal facilitation, intention, perceived behavioural control, and occupation as predictors of providing physical activity advice

Step	Predictor	***R***^**2**^	**Δ*R***^**2**^	*β*	*B*	*SE*	*p*	95% CI *B *Coefficient	
								**Lower**	**Upper**

1		0.48					<0.01		
	Intention			0.36	0.09	0.03	0.01	0.02	0.15
	PBC^1^			0.05	0.01	0.04	0.73	-0.06	0.09
	Occupation			0.49	0.48	0.12	<0.01	0.25	0.72
									
2		0.54	0.06				0.03		
	Intention			0.26	0.06	0.03	0.07	-0.004	0.13
	PBC^1^			-0.05	-0.01	0.04	0.73	-0.09	0.06
	Occupation			0.47	0.47	0.11	<0.01	0.24	0.70
	Goal Facilitation			0.30	0.10	0.05	0.03	0.01	0.19
									
3		0.62	0.08				<0.01		
	Intention			0.28	0.07	0.03	0.03	0.01	0.13
	PBC^1^			-0.15	-0.04	0.04	0.25	-0.11	0.03
	Occupation			0.43	0.43	0.10	<0.01	0.22	0.64
	Goal Facilitation			0.28	0.09	0.04	0.03	0.01	0.18
	Goal Conflict			-0.31	-0.07	0.02	<0.01	-0.12	-0.02

*Note*. Occupation reference category = GPs

^1^Perceived behavioural control

A secondary multiple linear regression was run to test the TPB hypothesis that attitude, subjective norm, and/or PBC are predictive of intention. Controlling for occupation, the predictors accounted for 46.0% of the variance in intention, with attitude (*β *= 0.45, *p *= 0.005) and perceived behavioural control (*β *= 0.33, *p *= 0.036) as significant predictors, thereby supporting the TPB hypothesis.

## Discussion

### Main findings

This exploratory study demonstrated the utility of goal facilitation and goal conflict for predicting the reported provision of PA advice by primary care health professionals, beyond intention and PBC from the TPB. Social cognition models and other theories applied in implementation science tend to focus on a single behaviour in isolation from the other behaviours performed in a given context. The present study presents a novel theoretical approach to understanding health professional behaviour. The novelty of this approach lies in the explicit consideration for the alternative behaviours that health professionals engage in and how these are perceived to facilitate and conflict with a particular clinical behaviour. The present study, while exploratory, shows that other clinical behaviours are perceived to help or hinder a given health professional behaviour and that such perceptions predict the reported provision of PA advice beyond PA advice-specific intention and PBC.

The potential of this approach is supported by testing the predictive utility of novel constructs against evidenced theory-based predictors of behaviour (such as those in the TPB). Given the preponderance of theories and theoretical constructs in the literature, the utility of novel constructs for predicting behaviour should be tested against existing theory [24]. Such tests promote theory development and move towards identifying a parsimonious set of constructs which each contribute independently to the prediction of behaviour.

The present study appears to be the first prospective study using and extending the TPB to predict the provision of PA advice by health professionals in primary care. It is also among the few which prospectively measures health professional behaviour, and thus heeds current calls from the literature for such longitudinal designs [10]. Despite relatively strong intention and PBC over giving PA advice, health professionals who perceived their competing GDBs as helpful and not hindering reported giving PA advice to more patients. The findings extend the existing evidence base for the utility of goal facilitation and goal conflict beyond motivational variables such as intention and PBC.

### Predicting health professional behaviour from goal facilitation and goal conflict

The significant effects of goal facilitation and goal conflict on behaviour beyond intention and PBC were equivalent in magnitude to intention's effect on behaviour. GDBs perceived to help and hinder providing PA advice thus aid in predicting how many patients will be given PA advice to the same extent as a health professional's intention to do so. This finding highlights the importance of considering the wider context of multiple goal pursuit in clinical practice. A core assumption in the TPB is that constructs in the theory sufficiently account for all effects on behaviour [25]. While this assumption argues for the necessity of including factors such as intention and perceived control when predicting behaviour, the present study suggests that these factors may be necessary but not sufficient.

The lack of association between goal conflict and goal facilitation themselves further supports the evidence suggesting that goal conflict and facilitation are distinct constructs that predict behaviour in different ways. Also, while associated to PBC, goal conflict and goal facilitation predicted behaviour over and above PBC, lending support to the notion that control constructs in the TPB do not sufficiently capture barriers and facilitators [13].

The current study replicates previous findings in the literature that goal facilitation predicts behaviour over and above intention and PBC [19], and extend the findings to a sample of healthcare professionals. The replication of this finding in a different population, context, and behaviour further supports the utility of goal facilitation as a predictive construct of behaviour above and beyond the TPB. That goal conflict predicts behaviour over and above intention and PBC differs from other studies that did not find goal conflict to predict preventive health-related behaviour [18-20]. The present study was explicitly conducted within a population whose pursuit of multiple goals is characteristically time-constrained, which may explain the difference in results compared to the aforementioned studies. Goal conflict may be more readily perceived in such contexts than in studies that ask participants about the goal conflict they perceive across the scope of their everyday life. This idea is supported by the observation that goal conflict results in the present study are consistent with those from studies in other constrained contexts [16,17]. These findings may help to bring some clarity to the equivocal nature of the evidence supporting goal conflict's role in predicting behaviour.

Constraining the context of multiple goal pursuit may have led to increased opportunity for GDBs to influence one another and thus be perceived as facilitating and conflicting with giving PA advice. Furthermore, the simplified measures of goal facilitation and goal conflict, compared to more elaborate cross-impact matrices previously used [19], may have contributed to larger effect sizes.

Mean levels of perceived conflict were moderate in the current study, suggesting that health professionals perceived that the negative influence of their competing GDBs, while present, was not particularly strong. This may be an indication of health professionals' capacity to effectively self-regulate their multiple GDBs such that despite resource constraints they manage to provide appropriate advice to some patients. However, the negative relationship between goal conflict and behaviour rather suggests that the more that participants perceived their competing GDBs as taking time away from giving PA advice, the fewer patients received PA advice.

### Implications for theory and practice

In their review of the effectiveness of guidelines for changing health professionals' behaviour, Grimshaw and colleagues argued for the need for testing and developing theory [26]. This study heeds these authors' suggestions by taking an integration approach to theory development. Although the TPB is among the models with the best predictive utility, its isolated focus on a single behaviour segregated from others has limited ecological validity for understanding behaviour in contexts where other GDBs are also performed. Although parsimonious, the TPB's evidenced lack of sufficiency has implications which extend beyond the predictive aims of the current study. Health professionals often report strong intention, perceived behavioural control, positive attitude, and a strong normative influence [27,28], and yet gaps between evidence and practice persist. Identifying additional predictors of behaviour beyond those in the TPB that are amenable to change may supplement efforts directed towards implementing clinical practice guidelines. This exploratory study demonstrates that goal conflict and facilitation can be such factors while also addressing limitations to the model. The present study suggests that further consideration should be given to how the existing GDBs being performed by health professionals might influence the performance of guideline-recommended behaviours being implemented. Future research is needed to test whether methods of optimising goal relationships--such as planning, shielding, and deferring the pursuit of other goals [29]--can change behaviour. However, the current study also highlights a point ignored by single-behaviour approaches: changing an existing behaviour, or introducing a new behaviour, may be influenced by the existing system of goal pursuit. Predispositions towards pursuing existing (potentially competing) goal-directed behaviours may help or hinder whether a behaviour is integrated into a goal system and pursued over time. Furthermore, promotion of a particular goal-directed behaviour may also have consequences for the existing goal system. While this was not tested in the current study, future research should consider how a focal GDB is perceived to help or hinder health professionals' other GDBs.

### Recruitment challenges and the use of single item measures

Despite a relatively small sample size, this study detected statistically significant effects because their magnitude was large. Although their confidence intervals may be wide, the effect sizes we found can help to inform sample size calculations for future research. Ideally, evidence-based recommendations for increasing responses rates should be used at all phases of data collection when feasible. We utilised many evidence-based methods of promoting questionnaire completion at follow-up, such as printing questionnaires in colour, sending questionnaires using recorded delivery, using shorter questionnaires, and including stamped return envelopes [30,31]. However, besides using short questionnaires, pragmatic constraints limited our ability to use additional techniques at baseline.

Small sample sizes are not uncharacteristic of theory-based studies with health professionals. Of the 14 prospective studies testing the TPB in health professionals identified by Godin *et al*.'s [10] systematic review, seven had sample sizes of 50 participants or less. Furthermore, many of the reviewed studies using postal questionnaires to collect data reported response rates of less than 25%. This underscores the recruitment challenges involved in conducting theory-based research with busy health professionals. These challenges are not new. Indeed, we expected a degree of attrition, and this was among the primary justifications for measuring constructs using single items. We kept the questionnaire short to promote a higher response and to reduce participant burden.

By convention, TPB studies typically assess constructs using multiple items and report an index of internal consistency, but such operationalisations do not address issues of validity. Multi-item measures used to assess intention tend to vary a single word in each item, often using words with similar but not identical meaning, assuming that they tap the same construct. While this may promote a high Cronbach's alpha, some wording reflects measures of demonstrably distinct constructs and may be theoretically unjustifiable. For example, a prototypical intention item is worded: 'I intend to do × behaviour in Y context at Z time.' However, additional items using similar wording such as 'I want,' 'I expect,' and 'I plan to' are commonly recommended intention items, despite being arguably related to separate constructs: 'I want' measures desire [32], 'I expect' measures behavioural expectation [33], and 'I plan to' can be viewed as a facet of a post-intentional planning measure [34]. The single item measures used in the present study allowed us to circumvent this issue.

The quality or presence of psychometric properties of predictors of health professional behaviour does not appear to be an effect modifier of the magnitude of the relationship between predictors and behaviour [10]. Scores on single items measures may be associated with behaviour to a similar magnitude as scores from multi-item measures, as we observed.

The present study was exploratory and serves to demonstrate that, given the consistently observed good psychometric properties of standard items across numerous studies, single items might be considered as an alternative to multi-item measures. Observed means and standard deviations from single items were consistent with other studies using composite scores based on multiple items [27,35]. In addition, the amount of variance in intention and behaviour accounted for (46% and 48%) was in line with mean frequency-weighted *R*^2 ^observed across TPB studies reported by Godin *et al*.'s review [10] (59% and 35%, respectively). Despite the limitations of single item measures, this study shows that scores based on such items can be effectively used in multiple regression-based analyses, and means, standard deviations, and effect sizes are similar to those garnered from composite scores based on multi-item scales.

### Limitations and future research

The measure of behaviour involved a two-week retrospective self-report, assessed six months after baseline. The two-week time period was selected to maximise the opportunity that health professionals would have seen patients, and thus had the opportunity to give them PA advice while providing a reasonable length of time for recalling such behaviour. While the self-reported behaviour was subject to recall bias, such measures can often be worded to more closely correspond to the predictive factors under study than objective measures of health professional behaviour [36]. Future research is nevertheless required using objective measures of behaviour with strong correspondence to measures of the predictive constructs. However, objective measures of provision of PA advice would require health professionals to reliably code the provision of advice in medical records, which may itself be influenced by the degree of competing goals vying for time. The exploratory nature of the present study provides an argument for a need for replication in other settings and health professional behaviours.

While the sample was randomly selected, the observed low response rate suggests that respondents might be a self-selected group. It may be the case that the sampled health professionals were those with sufficiently low goal conflict to have time to complete the questionnaire, while the non-respondents had higher goal conflict, which may have contributed to their non-response. This further underscores the relevance of goal conflict. Future research should aim to maximise response rates using evidenced methods [30] and assess the generalisability of the sample against population demographic variables besides those reported in the present study (*i.e*., sex).

Although single item measures move away from idiosyncratic measurement options taken in other studies [15,18,19], such measures may have simplified the typically more elaborate assessment of goal conflict and goal facilitation.

It is not clear to what extent goal conflict and facilitation vary over time in health professionals. Future research could consider whether the stability of goal conflict and facilitation might moderate the relationship between these factors and behaviour.

Finally, cross-sectional and prospective analyses preclude causal links from being tested. On the basis of the predictive evidence in support of goal facilitation and goal conflict, future research should test whether targeting these constructs for change in an intervention leads to behaviour change.

## Conclusion

The present study demonstrated that the strength with which primary care health professionals perceive their other GDBs to facilitate and conflict with them giving PA advice predicts how often they report providing such advice, over and above the TPB. Considering the perceived influence of other behaviours performed in a clinical consultation may help to better understand the provision of evidence-based care.

## Competing interests

The authors declare that they have no competing interests.

## Authors' contributions

This study was conceived by JP, JJF, NCC, and FFS. The study was run by JP. Data handling and analyses were conducted by JP. JP led the writing of this paper and all authors commented on drafts and approved the final version.
